# Clinical Indicators for Primary Cranial CT Imaging after Mild Traumatic Brain Injury—A Retrospective Analysis

**DOI:** 10.3390/jcm12103563

**Published:** 2023-05-19

**Authors:** Andreas Sakkas, Christel Weiß, Marcel Ebeling, Frank Wilde, Sebastian Pietzka, Qasim Mohammad, Oliver Christian Thiele, Robert Andreas Mischkowski

**Affiliations:** 1 Department of Cranio-Maxillo-Facial-Surgery, University Hospital Ulm, 89081 Ulm, Germany; 2 Department of Cranio-Maxillo-Facial-Surgery, German Armed Forces Hospital Ulm, 89081 Ulm, Germany; 3 Medical Statistics and Biomathematics, University Medical Centre Mannheim, Heidelberg University, 69167 Mannheim, Germany; 4 Institute for Diagnostic and Interventional Radiology, Ludwigshafen Hospital, 67063 Ludwigshafen, Germany; 5 Department of Cranio-Maxillo-Facial-Surgery, Ludwigshafen Hospital, 67063 Ludwigshafen, Germany

**Keywords:** intracranial hemorrhage, cranial computer tomography, mild traumatic brain injury, guidelines

## Abstract

The primary aim was to determine the clinical indicators for primary cranial CT imaging in patients after mild traumatic brain injury (mTBI). The secondary aim was to evaluate the need for post-traumatic short-term hospitalization based on primary clinical and CT findings. This was an observational retrospective single-centre study of all the patients who were admitted with mTBI over a five-year period. Demographic and anamnesis data, the clinical and radiological findings, and the outcome were analyzed. An initial cranial CT (CT0) was performed at admission. Repeat CT scans (CT1) were performed after positive CT0 findings and in cases with in-hospital secondary neurological deterioration. Intracranial hemorrhage (ICH) and the patient’s outcome were evaluated using descriptive statistical analysis. A multivariable analysis was performed to find associations between the clinical variables and the pathologic CT findings. A total of 1837 patients (mean age: 70.7 years) with mTBI were included. Acute ICH was detected in 102 patients (5.5%), with a total of 123 intracerebral lesions. In total, 707 (38.4%) patients were admitted for 48 h for in-hospital observation and six patients underwent an immediate neurosurgical intervention. The prevalence of delayed ICH was 0.05%. A Glasgow Coma Scale (GCS) of <15, loss of consciousness, amnesia, seizures, cephalgia, somnolence, dizziness, nausea, and clinical signs of fracture were identified as clinical factors with significantly higher risk of acute ICH. None of the 110 CT1 presented clinical relevance. A GCS of <15, loss of consciousness, amnesia, seizures, cephalgia, somnolence, dizziness, nausea, and clinical signs of cranial fractures should be considered absolute indicators for primary cranial CT imaging. The reported incidence of immediate and delayed traumatic ICH was very low and hospitalization should be decided individually considering both the clinical and CT findings.

## 1. Introduction

Traumatic brain injury is a common etiology of admission to emergency departments, with an economic impact on health systems [1]. Mild traumatic brain injury (mTBI) is defined by loss of consciousness for less than 30 min, amnesia for less than 24 h, psychiatric alteration, or focal neurological deficits, and an initial score of 13–15 on the Glasgow Coma Scale (GCS) [2,3,4].

Identification of high-risk minor head trauma patients still appears challenging for decision making by emergency physicians. Multiple validated clinical decision rules have been established to standardize and increase the efficiency of CT indication in patients after mTBI [5,6,7,8,9,10]. The Canadian CT Head Rule (CCHR) is the most common and has been found to be highly sensitive for detecting intracranial lesions; however, its specificity varies widely [10,11,12,13]. Other rules, such as the New Orleans criteria, the NICE, the Scandinavian rules, and NEXUS-II, based on different clinical and demographic indicators for imaging, have also demonstrated their validity and generalizability [9,14]. Antithrombotic therapy is an essential risk factor for the increased rate of traumatic intracranial pathologies [5,8,15,16,17]; however, most of the proposed decision rules are not valid for this patient cohort [18,19,20,21,22,23]. Delayed ICH can vary between 0.1% and 7% and the development of secondary deterioration is possible up to several weeks after the initial injury [5,24,25]. Consequently, no standard consensus exists regarding the appropriate primary treatment of patients with mTBI, which remains subject to the physician’s individual judgment [6,16,17,18,19,26,27,28,29].

Some hospitals follow protocols for the initial cranial CT scan and extensive monitoring under hospitalization with repeat imaging, if necessary, while others discharge asymptomatic patients after a negative CT scan [18,30,31,32]. Recommendations for hospitalization and repeat CT scan in patients with comorbid factors, such as antithrombotic therapy, vary at an international level [5,6,17,26]. Furthermore, the evaluation of different protocols regarding repeat CT scans and the hospitalization of asymptomatic patients has concluded that such a routine concept is obsolete [33,34]. The necessity of repeat CT scans in patients with an initially diagnosed ICH has also been questioned due to insignificant clinical relevance [30,32].

Depending on the structure of the emergency department, mTBI patients are attended by physicians with different specialties. In level I trauma centers, emergency physicians or neurosurgeons usually attend these cases primarily, while in other hospitals, the initial assessment is made by general surgeons, oral, and maxillofacial surgeons, or otorhinolaryngologists. Since various investigations on mTBI management have been published, especially from emergency medicine clinics, we acknowledge the lack of data from oral and maxillofacial surgery clinics.

The primary aim of this study was to determine the clinical indicators for primary cranial CT imaging in patients following mTBI over five consecutive years. The secondary aim was to estimate the necessity for post-traumatic in-hospital observation regarding the primary clinical and radiological findings.

## 2. Materials and Methods

### 2.1. Patient Collection

This retrospective, single-center study reviewed the medical records of all the patients who were referred to an oral and maxillofacial surgery clinic following an mTBI between January 2016 and December 2020. The records were retrieved from the hospital’s electronic database. Ethical approval for this research was obtained from the ethics committee of the chamber of physicians in Rhineland-Palatine, Mainz, Germany, in the context of a large retrospective trauma data evaluation with different aims and variable scientific issues (approval number: 2018-13524, approval date: 24 July 2018) [3,4].

Patients who met the following inclusion criteria were enrolled: (1) age, (2) head trauma, (3) a GCS score of 13–15 at admission, (4) loss of consciousness for less than 30 min, and (5) amnesia less than 24 h. The exclusion criteria were: (1) no cranial CT imaging at admission, (2) a GCS score of <13, (3) loss of consciousness for 30 min or longer, (4) amnesia for 24 h or longer, and (5) incomplete medical records [3,4].

### 2.2. Patient Screening

The study collective included patients with head trauma and any soft or hard tissue injury in the craniofacial area, or cognitive alteration. The presence or absence of ICH defined the CT result as positive or negative, respectively. A delayed ICH was defined as a new intracranial lesion on the CT scan, occurring within 2 weeks after the initial imaging and without secondary head injury.

Our standard clinical protocol included clinical assessment and an initial cranial CT scan (CT0) for all patients. CT of the midface, mandible, and/or the cervical spine was additionally performed in patients with suspected post-traumatic fractures of the midface, mandible, and cervical spine after clinical examination. Only patients admitted for in-hospital observation underwent laboratory tests. During the observation period, the mental status of the patient was evaluated every hour. A repeat cranial CT scan was performed in case of alterations in their neurological status. Neurological deterioration was defined as a decrease in the GCS score, a decrease in the level of consciousness, or the development of focal neurological deficits, such as worsening cephalgia, nausea, vomiting, vision changes, or dizziness [3,4]. Control CT scans of patients with positive CT0 findings were performed within 6–8 h after the initial scan. In the case of clinically insignificant CT1 results, patients were either discharged or other concomitant traumatic injuries of the facial skeleton were surgically addressed. Unstable patients at the initial examination were admitted to the intensive care unit (ICU) for primary monitoring and stabilization. Any necessary neurosurgical interventions were performed in the neurosurgery department of a nearby hospital after telemedical consultation, due to the absence of a neurosurgery unit in the center [3,4].

### 2.3. Data Collection

All the cranial CT scans indicated by the attending resident of the oral and maxillofacial surgery after the clinical examination and interpreted by board certified radiologists were collected. All the radiological findings related to an acute intracranial pathology (intracranial hemorrhage, subdural hematoma, epidural hematoma, subarachnoid hemorrhage, and hemorrhagic contusion) were documented. After consultation with the radiologic colleagues, we defined “intracranial hemorrhage” as non-trauma resulting intraparenchymal hemorrhage, e.g., in cases of pre-traumatic ischemic stroke, in order to differentiate from the trauma resulting “hemorrhagic contusions”. Traumatic lesions of the scull, midface, and mandible were also recorded. The clinical information not included in the emergency department report was considered negative.

All patients were anonymized before data analysis. The data were collected from patients’ hospital charts and included the patient’s age and gender; antithrombotic medication; cause of the trauma; the GCS score at initial examination; clinical examination findings indicating a cranial CT (loss of consciousness, amnesia, vomiting, headache, somnolence, dizziness, nausea, seizures, clinical signs of cranial fracture); traumatic intracranial lesions in the CT0 and CT1 scans; concomitant hard and soft tissue injuries of the scull and facial skeleton; and treatment in terms of discharge or admission (admission at the ICU or at the ward, duration of the hospitalization, neurosurgical intervention, in-hospital mortality). Regarding the cause of injury, we defined cases of ground-level fall, fall from a height, stair fall, traffic accident, bicycle accident, epileptic fall, and violence as cases not related to previous syncope or alcohol consumption.

### 2.4. Statistical Analysis

After electronic data centralization using Microsoft Excel software, statistical analysis was performed using SAS^®^, release 9.4 software (SAS Institute Inc., Cary, NC, USA). The patient’s age was expressed as mean and standard deviation (SD) and as a categorical variable (more or less than 65 years). Nominal data were expressed as absolute values (*n*) and relative incidences (%). Age >65 years, gender, antithrombotic treatment, and cause of injury were considered as patient-related risk factors. A GCS score of <15 and clinical features, such as loss of consciousness, headache, amnesia, vomiting, clinical signs of cranial fracture, and other neurological signs, were considered as trauma-related risk factors. The potential association between the investigated risk factors and the development of ICH was evaluated using chi-square tests. Fischer’s exact tests were used when the requirements for a chi-square test were not fulfilled, e.g., for smaller subgroups. The impact of a decreasing GCS score on the development of ICH was estimated using the Cochran–Armitage trend test. For the evaluation of the inter/intra-rater reliability for the categorical CT1 findings (“increasing”, “decreasing”, “constant”, “new”), McNemar’s test was applied and Cohen’s k-coefficient was calculated. The results were presented as tables and bar charts.

## 3. Results

### 3.1. Demographic Distribution

**Figure 1:**
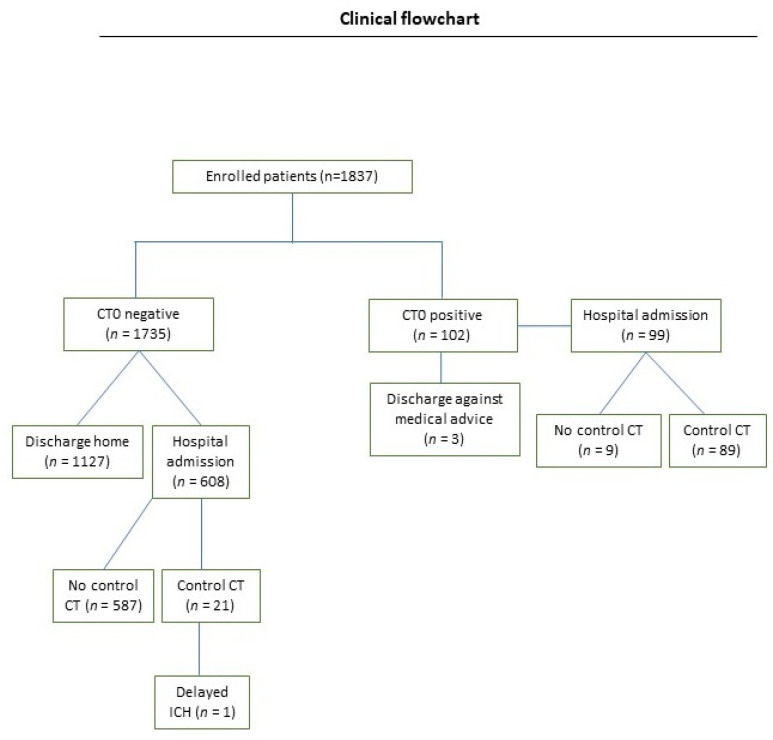
Study flowchart of patients with mTBI. Abbreviations: CT: computed tomography.

In total, 1837 patients with mTBI were included in the analysis (Figure 1). There were more males (*n* = 1016, 55.3%) than females (*n* = 821, 44.7%) (male to female ratio = 1.23:1). The patient’s age at the time of injury ranged from 21 to 106 years, with a mean age of 70.7 years (SD = ±21.10).

A ground-level fall was the most common cause of injury (*n* = 941, 51.2%). The average GCS score at presentation at the hospital was 15 in 91.1% of the patients, followed by 5.8% of the patients with a GCS of 14 and 3.1% of the patients with a GCS of 13. Neurological symptoms at the initial examination were present in 9.9% (*n* = 183) of the patients. Amnesia was the most endorsed symptom (*n* = 322, 17.5%), followed by loss of consciousness (*n* = 261, 14.2%), and cephalgia (*n* = 230, 12.5%). Clinical signs of cranial fracture were present in three (0.1%) patients. Contused lacerated wounds occurred in 61.1% of patients. Table 1 summarizes the cohort’s demographic features, clinical and radiological findings, and the outcome.

### 3.2. Antithrombotic Agents

There were 380 (54.5%) patients using antiplatelet agents, among them acetylsalicylic acid was the most common (96%). Eighty-eight patients (4.7%) were using vitamin K antagonists and 188 patients (10.2%) were undergoing DOAC therapy; among them rivaroxaban (44.4%) and apixaban (41.4%) were most prevalent. Six patients (0.3%) were taking heparin and 34 patients (4.8%) were undergoing double antithrombotic therapy [3].

### 3.3. CT Findings and Patient Outcome

One hundred and two patients (5.5%) had acute traumatic ICH, among them 39 (38.2%) were using antithrombotic medication. Twenty-one patients presented more than one traumatic lesion and a total of 123 radiologically detected intracerebral lesions were documented: 36 subdural hematomas, 9 epidural hematomas, 37 subarachnoid hemorrhages, 19 trauma-resulted contusion hemorrhages, and 22 non-trauma resulting intracranial hemorrhages. A skull fracture was detected in 1.0% of the study collective. Midface and mandible trauma were diagnosed in 60.7% and 45.4% of the patients, respectively. The C-spine of 107 patients also identified 13 cases (12.14%) of cervical spine trauma (Table 1).

**Figure 2:**
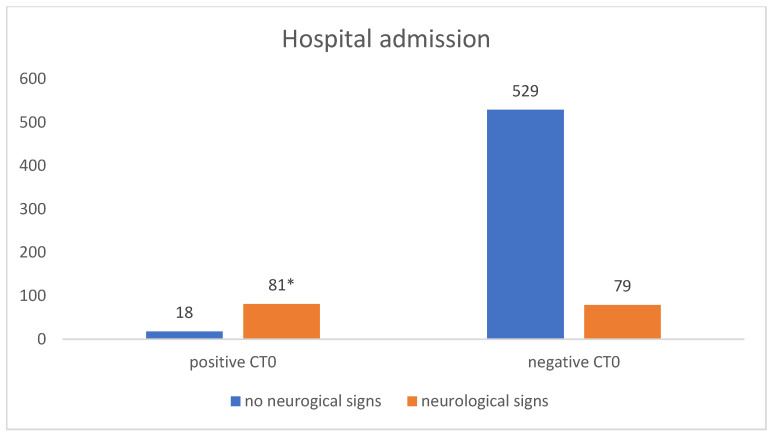
Presence of neurological signs in correlation with the CT0 findings in patients admitted for in-hospital observation. Abbreviations: CT0: initial computed tomography. * chi-square test: *p* < 0.00001.

Ninety-nine out of 102 (97.1%) patients with acute ICH were admitted for in-hospital observation (median length of stay: 7 days, SD = ±5). Three patients refused hospitalization and were discharged after receiving information about the risks and complications and providing their written consent. One of them presented a subarachnoid hemorrhage, one a subdural hematoma, and one a linear cranial fracture without clinical relevance. Most patients with acute ICH were male (55.8%, *n* = 57/102) and older than 65 years (57.8%, *n* = 59/102), and the ICH was caused by a ground-level fall in 41.1% (*n* = 42/102) of cases. Eighteen out of the 102 (17.6%) patients presented no neurological symptoms during the initial examination. In total, 61.5% (*n* = 1130) of the patients were discharged home after initial CT imaging. Among them, 1127 patients had a negative CT. Hospital admission for further observation took place for 707 (38.4%) patients (median length of stay: 4 days, SD = ±5), among them 258 patients were using antithrombotic medication. Most of the inpatients admitted (*n* = 608; 85.9%) had no radiological alterations at the initial CT. Among them, only 12.9% (*n* = 79) presented neurological alterations. Among 99 inpatients with pathologic CT0, 81.8% (*n* = 81) presented neurological alterations (*p* < 0.00001) (Figure 2). In 14 of the 36 patients (38.8%) with acute ICH, no neurological symptoms were observed; however, they were admitted for further in-hospital observation.

**Figure 3:**
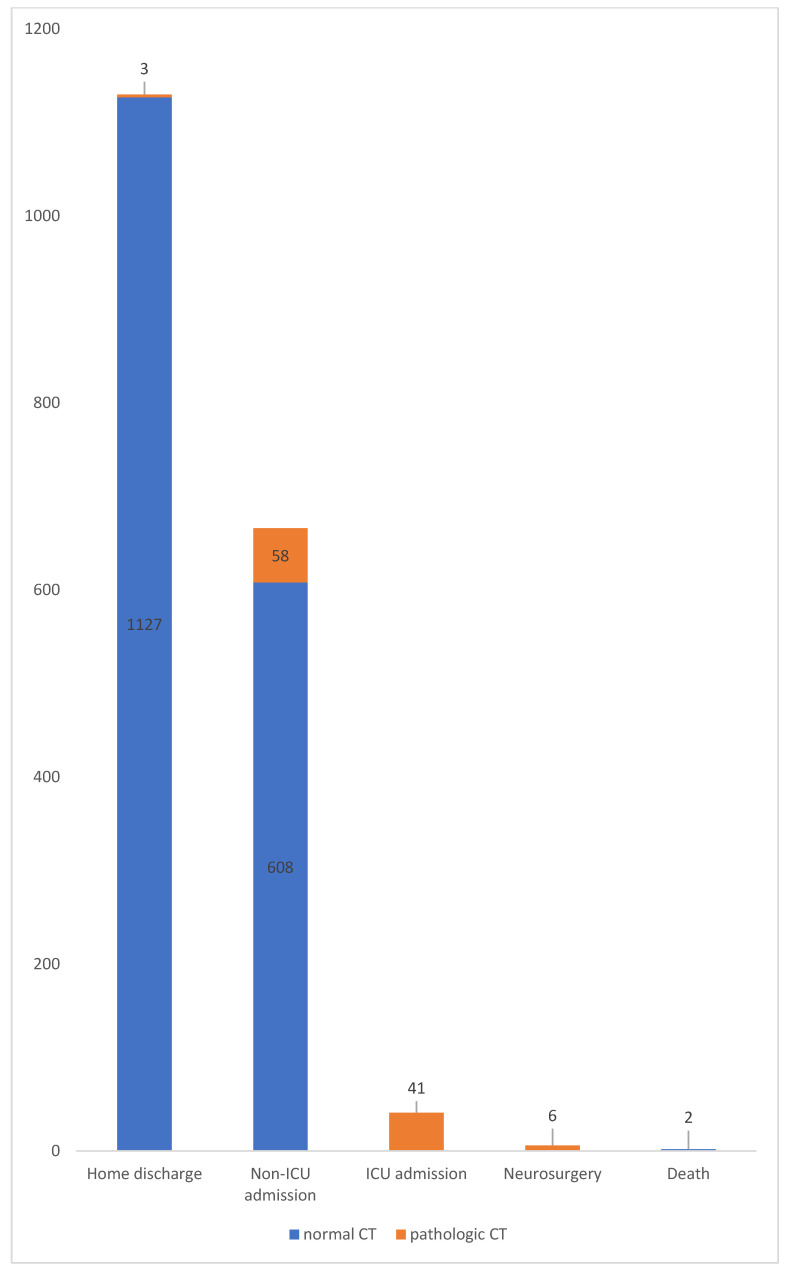
Distribution of patient management and outcome. Abbreviations: ICU: intensive care unit.

Forty patients (2.18%) were primarily admitted to the intensive care unit due to a reduction in their general condition, among them nine were undergoing antithrombotic therapy. Six patients required an urgent neurosurgical intervention. Overall, two (0.11%) patients died. The first patient was a 58-year-old male with a normal CT0 who reported cephalgia and dizziness at admission and was diagnosed with a severe cervical spine trauma. The second patient was a 94-year-old female without neurological alterations, diagnosed with an orbital floor fracture. Figure 3 shows the patients’ management and outcome.

**Figure 4:**
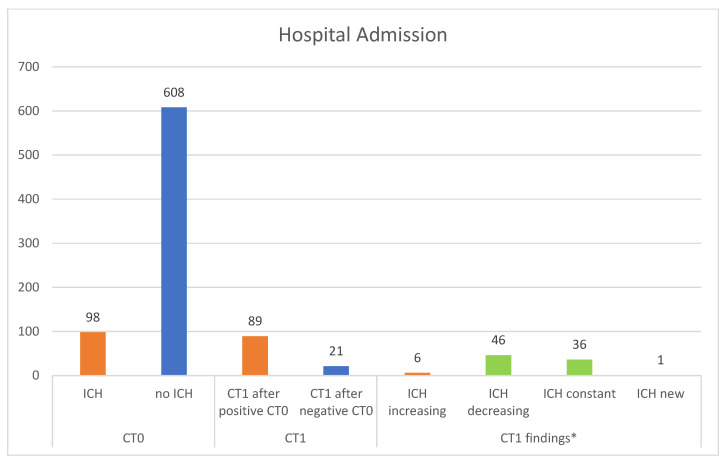
CT findings in patients admitted for in-hospital observation. * Cohen’s k-coefficient = 0.9700. Abbreviations: ICH: intracranial hemorrhage; CT0: initial cranial CT; CT1: repeat cranial CT.

One hundred and ten patients received a control CT scan (CT1) after 8 h. Among them, 89 patients had a positive CT0 and 21 patients had a negative CT0; however, they developed secondary neurological alterations during the hospitalization period. Delayed ICH was identified in one patient (0.05%). This 96-year-old female suffered a bicycle fall and was referred with an initial loss of consciousness, headache, amnesia, and a GCS score of 13 at the time of admission. A CT1 was performed due to secondary neurologic deterioration during the in-hospital observation period and a newly developed ICH was detected, however without clinical relevance. The intracranial lesion did not worsen in 82 patients, while intracranial bleeding increased in six patients, however, without indication of neurosurgical intervention (Figure 4). Regarding the patients who underwent a CT1, the number needed to screen (NNS) was 110, but this reduced to 21 when only the patients with a negative CT0 were considered (Table 2).

### 3.4. Multivariable Analysis

A GCS score of <15, a loss of consciousness, amnesia, seizures, cephalgia, somnolence, dizziness, nausea, and clinical signs of fracture were detected as the clinical factors significantly associated with an increased risk of acute ICH (*p* < 0.05). Vomiting and the use of antithrombotic medication were not associated with a higher prevalence of pathologic CT scans (Table 3). Considering the cause of the trauma, ground-level falls, falls from a height, traffic accidents, bicycle accidents, epileptic falls, and violence led to significantly higher rates of post-traumatic ICH (Table 4). A decreasing GCS (<15) was significantly related to the increased incidence of pathologic CT findings (*p* < 0.05) (Table 5).

## 4. Discussion

Mild traumatic brain injury has been sufficiently investigated by emergency physicians, neurosurgeons, and radiologists in the past. We conducted this study in a large trauma center in our region to evaluate the treatment outcome for patients who were primarily referred to an emergency department for oral and maxillofacial surgery following mTBI. The study represents a considerable patient collective that is commonly seen in emergency units internationally.

The study cohort had a similar age and gender to those in previous reports [5,6,8,28]. A ground-level fall was the most common cause of trauma, confirming the upcoming social problem of increasing age expectancy worldwide. This was also comparable with previous studies [1,5,19,24]. Most of our patients presented a GCS score of 15 at the time of initial examination. Amnesia was the most prevalent symptom followed by loss of consciousness, and headache. Except concomitant trauma of the midface and mandible, we also identified cervical spine trauma in 13 out of 107 patients who received a C-spine scan. However, the specific decision criteria applied for the C-spine indication in our collective could not be extracted from the available emergency reports. Thus, we suggest further studies comparing the clinical performance of the Canadian C-Spine Rule and the NEXUS Low-Risk Criteria to guide the use of cervical-spine radiography in patients with this pattern of trauma in our clinic.

We observed an acute ICH rate of 5.5%, likely approximate to the results in previous studies [5,6,8,16,28,35]. Subarachnoid hemorrhage was the most prevalent intracranial lesion, followed by subdural hematoma, and intracranial hemorrhage. We reported a very low delayed ICH of 0.1% without clinical relevance, findings that are in concordance with previous data, ranging from 0.13% to 6% [1,5,17,19,24,29,32,33,36,37,38]. In these studies, delayed intraparenchymal hemorrhages or extradural hematomas were the most commonly described cases. Considering also the rare, delayed development of subdural hematomas after minor head injury, follow-up appointments should always be arranged for clinical re-evaluation and patients should be informed accordingly in case of the delayed development of any kind of neurological alterations after discharge. The only case of delayed ICH in this study was recorded in a patient undergoing antiplatelet therapy. A statistical comparison of the delayed ICH rates between different antithrombotic agents or other independent clinical variables was not possible. Our findings confirm previous research that mTBI only rarely leads to delayed ICH and, even then, without clinical relevance. We recorded a mortality rate of 0.1% within the hospitalization period, significantly lower when compared to past reports [1,5,6,8,19,24].

The need for neurosurgical intervention based on the first CT scan was less than 1% of the mTBI [39,40]. According to Stipler et al., 0.8–4% of patients with either clinical or radiographic deterioration required delayed neurosurgical intervention after repeat CT imaging [39,40]. In our collective, six patients required an urgent neurosurgical intervention. An analysis of the risk factors for neurosurgical intervention was not possible in our study due to the small number of cases. However, we believe that an increased dimension of intracranial pathology in the repeat CT is not a determining factor for secondary surgery. Thus, we support decision making based on a conscientious clinical and neurologic examination, not only on CT imaging. Our results are in concordance with previous data stating that routine, repeat cranial CT cannot effectively identify patients who will require neurosurgical intervention after mTBI [39].

The clinical variables associated with increased CT pathology were loss of consciousness, amnesia, seizures, cephalgia, somnolence, dizziness, nausea, and clinical signs of cranial fractures. These characteristics can be easily derived from a structured initial examination, helping the clinical practice through risk stratification and indicating a primary cranial CT [8]. Wu et al., also stated that intracranial injury was significantly more probable in patients with an initial loss of consciousness [41]. Amnesia is considered as a medium risk factor for intracranial injury in the Canadian CT Head Rule [9,10]. In line with previous research, we also found that focal neurologic deficits, such as cephalgia, somnolence, dizziness, and nausea, are significant risk factors for a pathologic CT and should be considered as absolute indicators for primary CT imaging [14]. Seizures have not been sufficiently investigated in past studies; however, we consider the presence of seizures as a medium risk factor for CT abnormalities. A correlation between vomiting and positive CT scans was not found. Arab et al., and Borland et al., did not consider vomiting as a risk factor for minor head injuries in their studies, unlike Sadegh et al., and the American College of Emergency Physicians [13,14,42]. Two open skull fractures were detected among the 20 radiologically detected skull fractures in total. We believe that cases of open fractures should be classified as moderate or severe TBI in future studies, also considering the concomitant symptoms and comorbidities, since open fractures usually refer to a higher severity of injury. Regarding skull fractures, our results support primary CT imaging when cranial fractures are clinically present, but we also recommend a different TBI classification according to the radiological pattern of the fracture.

The clinical criterion that most accurately predicted CT pathologies was the GCS score, which contradicts the findings by Fournier et al., and Sadegh et al., [11,14]. A decreasing GCS score was a significant risk factor for increased pathologic lesions, which justifies CT imaging in the first place. We agree with Uchino et al., in recommending CT scans in patients with amnesia, even with a GCS of 15 [43]. However, we think that the GCS score may not always be a reliable triage tool, especially in patients with multiple neurological deficits or difficult verbal communication, resulting in inaccurate GCS score evaluation. The discharge of patients with a decreased GCS score should be based on an individual assessment, independent of the presence of an ICH. In our study, the GCS score detected pathologic CT scans with 29.41% sensitivity and 92.28% specificity, results similar to those of Lesko et al. [44]. This tool accurately detected five out of six patients who needed a neurosurgical intervention. The high specificity ensures that patients with a GCS score of 15 have a high probability of a normal CT. However, low sensitivity has to be considered, since significant positive findings could remain undetected, leading to increased CT use.

Although the predictor variables in this study were well standardized, no assessment of their interobserver agreement was made, and other potentially valuable features, such as alcohol or drug intoxication and high-energy trauma, were not assessed. We think that examination related to suspected intoxication of any kind could be unreliable for appropriate risk stratification in daily practice by presenting suspect neurological deficits, which otherwise would not exist without prior consumption. Therefore, we believe that intoxicated patients with a GCS score <15 should not automatically require a CT scan and suggest optional CT imaging due to these features.

An association between contused lacerated wounds and post-traumatic intracranial pathology was not detected, even when the wound incidence was high. Future studies with a standard use of the injury severity score could assist in the prediction of the risk of ICH according to the degree of soft tissue injury.

Our results did not confirm the widely-held belief that increased intracranial complications occur in patients using antithrombotic medication [6,8,28,45,46,47,48]. However, we did not aim to further investigate the correlation between the different antithrombotic agents with the incidence of ICH and the possible correlation with specific intracranial lesions. This should be the aim of future studies with a prospective design that involves homogenic groups of different antithrombotic medications.

Considering that the majority of previous studies referred to anticoagulated patients, our study investigated the necessity of repeat cranial CTs in mTBI patients with and without anticoagulation medication. Our results do not recommend routine, repeat cranial CT imaging, which is in line with previous studies [17,19,33,34,38,49,50,51]. We agree with Huang et al., in suggesting that both anticoagulated and non-anticoagulated patients should be treated similarly, since acute or delayed ICH usually does not have a clinical impact and very rarely requires neurosurgical intervention [5]. As Menditto et al., already stated, concomitant injuries, older age, and brain atrophy can be associated with delayed ICH, which are more likely than antithrombotic medication [32]. This strategy would decrease hospital costs and radiation exposure and improve patient satisfaction. The rate of secondary neurological alteration during the in-hospital observation period in our study was 1.1%. One of these 21 patients developed a delayed ICH, however, without clinical relevance, presenting a very low needed to screen number of 21 for patients with a negative CT0. Taking this into consideration, we expressively suggest repeat CT scans in cases involving the development of secondary neurological symptoms during the in-hospital observation period or even after discharge.

Our study does not support routine 24–48 h hospital observation of mTBI patients. This applies also for patients undergoing antithrombotic treatment, in concordance with the conclusions of Campiglio et al. [1]. Patients should be admitted to hospital on an individual basis. We believe that patients with supratherapeutic INR levels, patients with severe injury mechanisms, patients without adequate home surveillance, and patients who do not understand the discharge recommendation should continue to be observed. The management of older patients should also be carefully evaluated to ensure the best outcome for this vulnerable cohort. We agree with Fuller et al., that older patients prefer to take risks and avoid lengthy hospital stays [3,4,6]. In such cases, patients and their families should be fully informed of the relative risk of late complications to ensure a safe discharge home.

The study findings represent a wide spectrum of characteristics and sites and can be easily applied to clinical practice. An important strength is that all the participants were included without drop-out. Nevertheless, we acknowledge some limitations. First, the study was limited to a single level I trauma centre, which could limit generalizability. Second, the observational, retrospective nature of the study could lead to documentation bias, especially on symptoms and clinical signs. Third, this study was conducted only on patients referred to our regional clinic for oral and maxillofacial surgery. Ambulances may preferentially send more severe cases to other specialist facilities or local hospitals, creating a biased representation of severity. Fourth, our study protocol did not investigate events after the discharge of the patients. Fifth, we included only participants with mild TBI so our results cannot be extrapolated to patients with severe TBI. Finally, the absence of a control group of patients without an initial CT scan could also limit our study results. Future well-designed, randomized controlled trials with a multi-institutional design should validate our findings for clinical practice.

## 5. Conclusions

Our study reported a very low incidence of immediate and delayed traumatic ICH in patients after mTBI. A GCS of <15, a loss of consciousness, amnesia, seizures, cephalgia, somnolence, dizziness, nausea, and clinical signs of cranial fractures are highly recommended clinical indicators for primary cranial CT imaging. These features should provide physicians with clear directions for CT indication in emergency departments without CT possibility. We encourage shared patient–physician decision making and recommend repeat cranial CT imaging for patients with secondary neurological deterioration. We do not support in-hospital observation without distinction between both patients with or without antithrombotic medication, and recommend individual decision making based on careful assessment of both the clinical and CT findings, however with appropriate discharge instructions and home surveillance. We support continuing medical education for decision support and encourage good communication between physicians and patients. Future prospective randomized controlled studies should validate our preliminary findings.

## Figures and Tables

**Table 1:** Baseline clinical characteristics and radiological findings for the overall study population after mild TBI.

Study Population	N	%
	1837	100%
Demographics		
Age ≥ 65 years	1062	57.81%
Age < 65 years	775	42.19%
Male	1016	55.31%
Female	821	44.69%
Cause of injury		
Ground-level fall	941	51.22%
Fall from height	21	1.14%
Stair fall	19	1.03%
Traffic accident	30	1.63%
Bicycle accident	116	6.31%
Epileptic fall	17	0.93%
Fall after syncope	154	8.38%
Violence	292	15.90%
Sport	20	1.09%
Fall after alcohol consumption	185	10.07%
Other mechanism	42	2.29%
GCS scale		
15	1673	91.07%
14	106	5.77%
13	58	3.16%
Clinical examination		
Loss of consciousness	261	14.21%
Headache	230	12.52%
Amnesia	322	17.53%
Vomiting	45	2.45%
Neurological signs	183	9.96%
Seizures	17	0.93%
Clinical signs of cranial fracture	3	0.16%
Contused lacerated wound	1141	62.11%
CT findings		
Subdural hematoma	36	1.96%
Epidural hematoma	9	0.49%
Subarachnoid hemorrhage	37	2.01%
Contusion hemorrhage (trauma resulting)	19	1.03%
Intracranial hemorrhage (non-trauma resulting)	22	1.20%
Galea hematoma	203	11.05%
Midface trauma	567	60.7% *
Mandible trauma	85	45.45% **
Cervical spine trauma	13	12.14% ***
Admission disposition		
Home	1131	61.57%
Non-ICU	666	36.25%
ICU	40	2.18%
Neurosurgery	6	0.33%
Death	2	0.11%

Abbreviations: N: number; GCS: Glasgow Coma Scale; ICU: intensive care unit. * refers to the total number of patients who received a midface CT scan (*n* = 934). ** refers to the total number of patients who received a mandible CT scan (*n* = 187). *** refers to the total number of patients who received a cervical spine CT scan (*n* = 107).

**Table 2:** Demonstration of the CT1 findings in patients with a positive or negative CT0.

	Frequency (n)	Percent (%)	Cumulative	Cumulative Percent (%)	NNS	95% CI
			Frequency (n)			
CT1 positive (total)	1	0.90%	1	0.90%		
CT1 negative (total)	109	99.10%	110	100	110	21–5000
CT1 positive (with CT0 negative)	1	4.70%	1	4.70%		
CT1 negative (with CT0 negative)	20	95.30%	21	100	21	5–834

Abbreviations: n: number; NNS: number needed to screen; CI: confidence interval.

**Table 3:** CT0 findings in correlation with the demographic and clinical variables after mild traumatic brain injury.

		Positive CT0 Findings 102 (5.55%)		Negative CT0 Findings 1735 (94.45%)		*p*-Value
Characteristics		n	%	n	%	
Gender	Male	57	5.61%	959	94.39%	* 0.9044
	Female	45	5.48%	776	94.51%	
Age	≥65 years	59	5.56%	1003	94.44%	* 0.9947
	<65 years	43	5.55%	732	94.45%	
GCS score	13/14	30	18.29%	134	81.71%	* <0.0001
	15	72	4.30%	1601	95.70%	
Loss of consciousness	Yes	39	14.94%	222	85.06%	* <0.0001
	No	63	4.0%	1513	96.0%	
Amnesia	Yes	31	9.63%	291	90.37%	* 0.0004
	No	71	4.69%	1444	95.31%	
Vomiting	Yes	3	6.67%	42	93.33%	** 0.7360
	No	99	5.52%	1693	94.48%	
Seizures	Yes	4	23.52%	13	76.48%	* 0.0122
	No	98	5.38%	1722	94.62%	
Cephalgia	Yes	32	13.91%	198	86.09%	* <0.0001
	No	70	4.36%	1537	95.64%	
Somnolence	Yes	9	16.36%	46	83.64%	** 0.0026
	No	93	5.22%	1689	94.78%	
Dizziness	Yes	29	16.48%	147	83.52%	* <0.0001
	No	73	4.39%	1588	95.61%	
Nausea	Yes	9	12.16%	65	87.84%	** 0.0189
	No	93	5.28%	1670	94.71%	
Clinical signs of cranial fracture	Yes	3	100%	0	0%	** 0.0002
	No	99	5.39%	1735	94.61%	
Antithrombotic medication	Yes	39	5.60%	657	94.40%	* 0.9407
	No	63	5.52%	1078	94.48%	

Abbreviations: n: number; %: percentage. * chi-square test. ** Fischer’s exact test.

**Table 4:** CT0 findings in correlation with the trauma mechanism after mild traumatic brain injury.

		Positive CT0 Findings 102 (5.55%)		Negative CT0 Findings 1735 (94.45%)		
Mechanism of Injury		n	%	n	%	*p*-Value
Ground-level fall	941	42	4.46%	899	95.54%	* 0.0367
Fall from height	21	6	28.57%	15	71.43%	** 0.0007
Stair fall	19	2	10.53%	17	89.47%	** 0.2849
Traffic accident	30	5	16.67%	25	83.33%	** 0.0225
Bicycle accident	116	13	11.21%	103	88.79%	* 0.0060
Epileptic fall	17	4	23.53%	13	76.47%	** 0.0122
Fall after syncope	154	11	7.14%	143	92.86%	* 0.3679
Violence	292	8	2.74%	284	97.26%	* 0.0221
Sport	20	0	0%	20	100%	* 0.6231
Fall after alcohol consumption	185	9	4.86%	176	95.14%	* 0.6667
Other mechanism	42	2	4.76%	40	95.24%	** 1.0000

Abbreviations: n: number; %: percentage. * chi-square test ** Fischer’s exact test.

**Table 5:** Correlation of the cranial CT findings and the initial GCS score.

		GCS = 13/14 164 (8.93%)		GCS = 15 1673 (91.07%)		
CT Findings	Total	n	%	n	%	*p*-Value
Intracranial hemorrhage	22	10	6.09%	12	0.71%	* <0.0001
Subdural hematoma	36	12	7.31%	24	1.43%	* <0.0001
Epidural hematoma	9	4	2.43%	5	0.29%	** 0.0054
Subarachnoid hemorrhage	37	9	5.48%	28	1.67%	* 0.0009
Contusion hemorrhage	19	6	3.65%	13	0.77%	** 0.0047
Depressed skull fracture	3	2	1.21%	1	0.05%	** 0.0224
Linear skull fracture	9	4	2.43%	5	0.29%	** 0.0054
Open skull fracture	2	2	1.21%	0	0%	** 0.0079
Basal skull fracture	6	5	3.04%	1	0.05%	** 0.0000
Galea hematoma	203	10	6.09%	193	11.53%	* 0.0340

Abbreviations: n: number; %: percentage; GCS: Glasgow Coma Scale. * chi-square test. ** Fischer’s exact test.

## Data Availability

The data presented in this study are available on reasonable request from the corresponding author.
